# Mortality of continuous infusion versus intermittent bolus of meropenem: a systematic review and meta-analysis of randomized controlled trials

**DOI:** 10.3389/fmicb.2024.1337570

**Published:** 2024-03-08

**Authors:** Ming-Ying Ai, Wei-Lun Chang, Chia-Ying Liu

**Affiliations:** ^1^Department of Pharmacy, Far Eastern Memorial Hospital, New Taipei City, Taiwan; ^2^Department of Internal Medicine, Far Eastern Memorial Hospital, New Taipei City, Taiwan

**Keywords:** continuous infusion, meropenem, mortality, resistant pathogens, bacterial eradication

## Abstract

**Background:**

Meropenem belongs to the carbapenem class, which is categorized as beta-lactam antibiotics. These antibiotics are administered in intermittent bolus doses at specific time intervals. However, the continuous infusion approach ensures sustained drug exposure, maintaining the drug concentration above the minimum inhibitory concentration (MIC) throughout the entire treatment period. This study aimed to find out the association between continuous infusions of meropenem and mortality rates.

**Materials and methods:**

We conducted a search of the PubMed/Medline, EMBASE, Cochrane Central, and ClinicalTrials.gov databases up to 14 August 2023. The six randomized controlled trials (RCTs) were identified and included in our analysis. The random-effects model was implemented using Comprehensive Meta-Analysis software to examine the outcomes.

**Results:**

Our study included a total of 1,529 adult patients from six randomized controlled trials. The primary outcome indicated that continuous infusion of meropenem did not lead to reduction in the mortality rate (odds ratio = 0.844, 95% CI: 0.671–1.061, *P* =0.147). Secondary outcomes revealed no significant differences in ICU length of stay (LOS), ICU mortality, clinical cure, or adverse events between continuous infusion and traditional intermittent bolus strategies of meropenem. Notably, we observed significant improvements in bacterial eradication (odds ratio 19 = 2.207, 95% CI: 1.467–3.320, *P* < 0.001) with continuous infusion of meropenem. Our study also suggested that performing continuous infusion may lead to better bacterial eradication effects in resistant pathogens (coefficient: 2.5175, *P* = 0.0138^*^).

**Conclusion:**

Continuous infusion of meropenem did not result in the reduction of mortality rates but showed potential in improving bacterial eradication. Furthermore, this strategy may be particularly beneficial for achieving better bacterial eradication, especially in cases involving resistant pathogens.

## 1 Introduction

Beta-lactam antibiotics, which are time-dependent antibiotics, possess a common structure known as the beta-lactam ring and are widely utilized for treatment of diverse bacterial infections (Bush and Bradford, 2016). Traditionally, these antibiotics have been administered as intermittent bolus doses at specific time intervals. These antibiotics typically exert their antimicrobial effects by binding to bacterial enzymes involved in cell wall synthesis (Zeng and Lin, 2013). The time-dependent killing property is reliant on the duration of the pathogen's exposure to the antibiotics. Extended or continuous infusion aids in overcoming the time-dependent nature of beta-lactam antibiotics (Tilanus and Drusano, 2023).

One strategy employed to optimize the effectiveness of beta-lactam antibiotics involves the utilization of continuous infusion, which prolongs the duration of the antibiotic bolus to 24 h. The continuous infusion approach ensures sustained drug exposure, maintaining the drug concentration above the minimum inhibitory concentration (MIC) during the entire treatment period (Shiu et al., 2013). Administering meropenem through continuous infusion can help maintain constant therapeutic levels, potentially improving bacterial eradication and reducing mortality rates. Additionally, previous studies have indicated that extended and continuous infusion strategies have the potential to lower the serum peak concentration, thereby minimizing the likelihood of adverse effects caused by drug toxicity (Cotner et al., 2017).

Meropenem, belonging to the carbapenem class of broad-spectrum beta-lactam antibiotics, is used for treating severe bacterial infections often caused by multidrug-resistant organisms (MDROs) in critically ill patients (Hellinger and Brewer, 1999). Although some previous studies have showed that continuous infusion of meropenem offers several advantages, including stable drug levels and reduced adverse effects, there are still some potential caveats. For example, continuous infusion necessitates special equipment, such as an infusion pump, to be administered continuously for 24-h, entails cost consideration, and requires additional nursing care to ensure the correct dosage is being administered. This may increase the overall cost for the hospital (Dunning and Roberts, 2015).

While previous hypotheses have suggested that continuous infusion could provide stable therapeutic levels and potential advantages for specific antibiotics, limited clinical evidence exists, especially regarding continuous infusion of meropenem. In this study, we aim to investigate the association between continuous infusion of meropenem and mortality rates.

## 2 Materials and methods

### 2.1 General guidelines

We adhered to the steps outlined in the recent edition of the PRISMA 2020 guidelines (Page et al., 2021) for conducting this meta-analysis. This study was registered with INPLASY under the registration number INPLASY 2023110035 (Ai et al., 2023) and was exempted from obtaining ethics review board approval and participant informed consent.

### 2.2 Data research and the identification of eligible studies

Two authors (MY, Ai and CY, Liu) independently conducted electronic searches in the PubMed, Embase Cochrane CENTRAL, and ClinicalTrials.gov databases using the keywords [continuous infusion AND (carbapenem OR meropenem)]. The search period covered from each database to the date of 4 August 2023. The gray literature was also considered in our study. However, the gray literature that was searched for in our study was all excluded during data extraction process because none of them were randomized control trials (RCTs).

Initially, the two authors responsible for the search screened the titles and abstracts of the identified studies for eligibility using the consensus process. Subsequently, a thorough screening of full texts was conducted.

### 2.3 Inclusion and exclusion criteria

The PICO (population, intervention, comparison, outcome) framework for the current meta-analysis is as follows: P: human participants, I: continuous infusion of meropenem, C: intermittent bolus of meropenem, and O: mortality.

The inclusion criteria include the following: (1) Enrolled human participants in RCTs. (2) RCTs comparing the mortality rate of continuous infusion and intermittent bolus of meropenem to treat infection.

The exclusion criteria: (1) NON-RCTs study. (2) Human participants were not enrolled. (3) RCTs that did not examine the outcome of mortality. (4) RCTs, but only investigated the mortality rate between extended infusion and intermittent bolus of any meropenem. (5) RCTs, but not investigated the mortality rate between extended infusion and intermittent bolus of other carbapenems (except meropenem). (6) RCTs investigated the mortality rate of extended infusion and intermittent bolus of other beta-lactam agents.

### 2.4 Methodological quality appraisal

To assess the methodological quality of the included studies, we utilized the Cochrane risk-and-bias tool for randomized trials (version 2, RoB 2, London, United Kingdom) (Sterne et al., 2019). This tool consists of six main domains for evaluating the study quality, including randomization, intervention adherence, missing outcome data, outcome measurement, selective reporting, and the overall risk of bias. Regarding the intervention adherence section of the RoB 2 tool, two options were available for literature assessment: intention-to-treat (ITT) and per-protocol (PP). In our research, we incorporated studies from randomized controlled trials (RCTs) that utilized both ITT and PP analyses.

ITT and PP analyses stand as pivotal methodologies in clinical trial research, each contributing distinct insights. The ITT approach encompasses all randomized participants, irrespective of their study completion or adherence to the protocol. This methodology reduces selection bias and upholds the advantages of randomization, thereby offering results that more accurately mirror real-world conditions. ITT analysis is vital for extrapolating results to a broader patient demographic, mirroring the variable adherence often seen in everyday clinical settings.

PP analysis is more selective, focusing solely on participants who adhered strictly to the study protocol until completion. This approach is key to gauging a treatment's efficacy in optimal conditions, thereby elucidating its maximum potential effectiveness. By evaluating only those who strictly followed the treatment plan, PP analysis provides a more precise estimate of the treatment's impact, albeit with less generalizability to the wider population.

In essence, while ITT analysis presents a realistic portrayal of treatment outcomes in typical clinical practice, PP analysis sheds light on the optimal efficacy of treatments under ideal conditions. Collectively, both analyses offer a thorough understanding of a treatment's effectiveness across various scenarios. However, all six studies included in our research have consistently presented outcomes derived from ITT analysis.

### 2.5 Primary outcomes

We compared the mortality rate between continuous infusion and intermittent bolus of meropenem. We also analyzed mortality trends in different subgroups, including meropenem, other beta-lactam agents, and different continuous infusion doses. The outcome was measured and quantified using odds ratios. The sensitivity test and publication bias were also evaluated.

### 2.6 Secondary outcomes

The secondary outcomes include clinical success/improvement, intensive care unit (ICU) mortality, length of ICU stay, and bacterial eradication rate, comparing continuous infusion with intermittent bolus administration of meropenem. Treatment-related serious adverse events were also analyzed in our study. In instances where cells had zero events, a value of 0.5 was substituted to facilitate calculations (Deeks et al., 2022). The outcome was measured and quantified using odds ratios. The sensitivity test and publication bias were evaluated.

The meta-regression was performed to evaluate the relationship between mortality and continuous infusion dose. The correlation between resistant pathogens and mortality or bacteria eradicated when continuous infusions were performed was also studied. The resistant pathogen was definite as the pathogen was resistant to carbapenem or culture susceptibility test results showed a meropenem MIC ≥ 1.5.

### 2.7 Data extraction and management

Data extraction from the evaluated studies was carried out by two independent authors (M-YA and C-YL). The extracted data included demographic information, study design parameters, details of continuous infusion and intermittent administration of meropenem, as well as primary and secondary outcome values.

### 2.8 Statistical analysis

Based on the variability in target populations across the included studies, we performed the current meta-analysis using a random-effects model implemented using Comprehensive Meta-Analysis software (version 3, Biostat, Englewood, NJ, United States). A two-tailed *p*-value of < 0.05 was considered statistically significant (Borenstein and Hedges, 2009).

A fixed-effect model assumes that the true effect of the intervention is the same in all studies (i.e., fixed across studies). It implies that any observed differences among study results are attributed solely to chance (random variability). The fixed-effect model operates on the strong assumption that intervention effects are identical across all studies. It is typically used when the studies are very similar in terms of participants, interventions, and outcomes. However, this model does not account for heterogeneity across studies.

The random-effects model does not assume a single true effect size but rather a distribution of effect sizes. It assumes that the effects follow a normal distribution and recognizes that differences in study results may be due to both chance and genuine variation in intervention effects. This model is more flexible and realistic in many scenarios, especially when there is an expectation of variability in intervention effects across studies. It is particularly useful when the studies in the meta-analysis are not homogenous in terms of populations, interventions, outcomes, or methodologies.

In our article, we consider that assuming identical intervention effects across various studies is generally implausible, barring exceptional cases where the intervention exhibits no effect whatsoever. This argument favors the adoption of the random-effects model in our study. By acknowledging and accommodating the inherent heterogeneity in meta-analyses, the random-effects model offers a more nuanced and potentially more accurate estimation of the average intervention effect.

To quantify the primary outcomes, Hedges' g and calculated 95% confidence intervals (CIs) were used. Effect sizes were categorized as small (*g* = 0.2), moderate (*g* = 0.5), and large (*g* = 0.8) based on Hedges' criteria (Hedges, 1981).

To assess the degree of heterogeneity among the studies, we examined *I*^2^ and Cochran's Q statistics. *I*^2^ values of 25, 50, and 75% were considered indicative of low, moderate, and high heterogeneity, respectively (Higgins et al., 2003).

We also performed meta-regression analyses to investigate the relationship between the mortality rate and the continuous infusion dose.

To ensure the robustness of this meta-analysis, sensitivity analyses were conducted using the one-study removal method to examine whether removing a particular trial resulted in a significant change in the summary effect size (Deeks et al., 2022).

Potential publication bias was assessed following the guidelines outlined in the Cochrane Handbook for Systematic Reviews of Interventions. Funnel plots and Egger's regression tests were used to evaluate the presence of public bias included in the studies (Page et al., 2022).

## 3 Results

### 3.1 Study identification and selection

**Figure 1:**
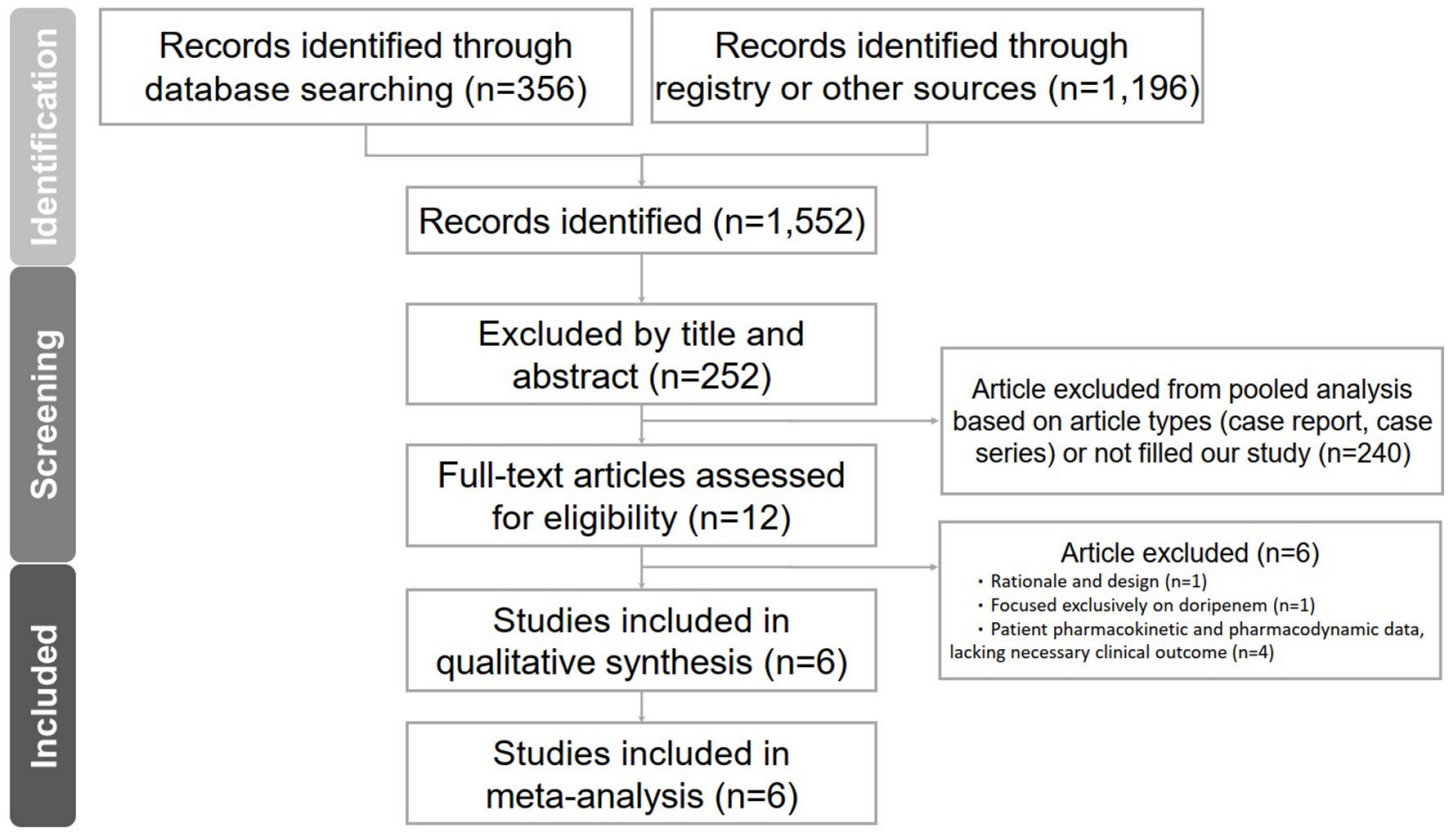
PRISMA flowchart.

**Figure 2:**
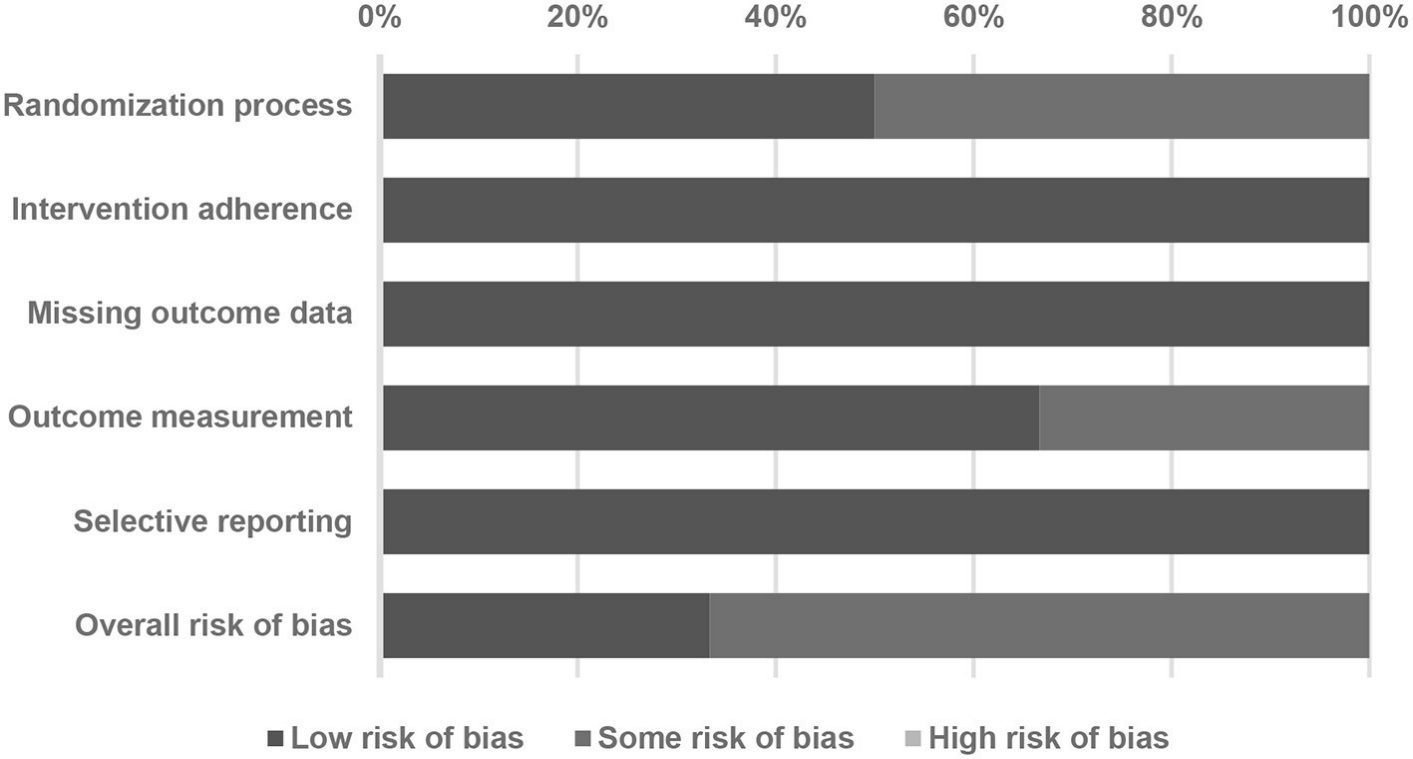
A summary of quality assessment of studies included in the meta-analysis using the Cochrane risk-of-bias 2 tool.

The PRISMA flowchart depicts the sequential process undertaken to identify and select studies for analysis (Figure 1). Initially, we conducted a comprehensive search of relevant databases using appropriate keywords and search terms. A total of six RCTs were included in our meta-analysis, as shown in Table 1 (Chytra et al., 2012; Dulhunty et al., 2013, 2015; Abdul-Aziz et al., 2016; Zhao et al., 2017; Monti et al., 2023). The results of the Cochrane RoB 2 assessment for methodological quality were also evaluated (Figure 2; Table 2). Our meta-analysis comprised six RCTs, involving a total of 1,529 adult individuals. All the studies included in our analysis focused on adult populations.

**Table 1:** A summary of the RCTs investigating the continuous infusion and intermittent bolus strategies among the enrolled participants.

**First author (year)**	**Country**	**Population**	**Sample size**	**Age (CI/BI)**	**APACHEII score (CI/BI)**	**Antibiotics**	**Meropenem Regimen**	**Resistant pathogen (%)**	**Study design**	**Infection type CI/BI (%)**	**Pathogen CI/BI (%)**	**Sensitivity analysis**	**SAE associated with study withdrawal**
Chytra (2012)	Czech	ICU	• CI: 120 • BI: 120	• 44.9 ± 17.8 • 47.2 ± 16.3	• 21.4 ± 7.9 • 22.1 ± 8.79	Meropenem	• CI: LD:2 g; MD:4 g QD • BI: 2 g Q8H	11.67^c^	RCT, open-label	• Respiratory: 55.0/50.8 • Abdominal: 19.2/25.8 • Uroinfection:9.2/5.0 • Bloodstream:8.3/9.2 • Soft tissue:4.2/5.0 • CNS:2.5/1.6 • Other:2.5/0.8	• *Klebsiella* spp.: 59.4/46.3 • *Acinetobacter* spp.: 7.5/11.1 • *Escherichia coli*: 5.7/8.3	MIC determination	Not happened in both groups
Dulhunty (2013)	• Australia • Hong Kong	5 ICUs	• CI:30 • BI:30	• 54 ± 19 • 60 ± 19	• 21 ± 8.6 • 23 ± 7.6	PTZ, Meropenem **Ticarcillin** • Clavulanate	• CI: 3 g QD • BI: 1 g Q8H	Not mention	RCT, double blind	• Respiratory: 36.8/43.2 • Bloodstream: 18.4/18.9 • Abdominal: 15.8/18.9 • Uroinfection: 7.9/5.4 • Soft tissue: 7.9/8.1 • CNS: 5.3/0 • Other: 2.6/0	Various	MIC determination	Not happened in both groups
Dulhunty (2015)	• Australia • Hong Kong • New Zealand	25 ICUs	• CI: 212 • BI: 220	• 64 (54–72) • 65 (53–72)	• 21 (17–26) • 20 (16–25)	PTZ, Meropenem Ticarcillin • Clavulanate	• ^b^CI: 3 g QD • ^b^BI: 1 g Q8H	Not mention	RCT, double blind	• Respiratory: 54.2/54.5 • Abdominal: 25.0/25.9 • Bloodstream: 8.0/8.2 • Uroinfection: 7.5/8.2 • Soft tissue: 6.1/8.2 • Other: 20.0/11.9	• Gram-positive: 27.5/25.6 • Gram-negative: 72.5/72.1	Not specified	Not happened in both groups
Abdual (2016)	Malaysian	2 ICUs	• CI: 70 • BI: 70	• 54 (42–63) • 56 (41–68)	• 21 (17–26) • 21 (15–26)	PTZ, Meropenem • Cefepime	• CI: LD: 1 g; MD: 3 g QD • BI: 1 g Q8H	Not mention	RCT, open-label	• Respiratory: 66/51 • Abdominal: 16/21 • Bloodstream: 6/9 • Uroinfection: 3/4 • Soft tissue: 9/10 • Other: 1/4	• Gram-positive: 20/33 • Gram-negative: 80/67	MIC determination.	Not happened in both groups
Zhao (2017)	China	ICU	• CI: 25 • BI: 25	• 68.0 ± 15.4 • 67.0 ± 12.2	• 19.4 ± 5.0 • 19.7 ± 5.9	Meropenem	• CI: LD:0.5 g; MD: 3 g QD • BI: LD: 1.5 g; MD: 1 g Q8H	31.81^c^	RCT	• Respiratory: 36.0/40.0 • Abdominal: 56.0/52.0 • Bloodstream: 20.0/12.0 • Uroinfection: 4/8 • Soft tissue: 4/0 • Other: 0/4	• *Escherichia coli*: 26% • *Pseudomonas aeruginosa*: 24% • *Klebsiella* spp.: 16% • *Acinetobacter* spp.: 12% • *Enterobacter* spp.: 4% • *Providencia* spp.: 2% • *Burkholderia*: 2%	MIC determination	Not mention
Giacomo (2023)	• Croatia • Italy • Kazakhstan • Russia	31 ICUs	• CI: 303 • BI: 304	• 65.5 (14.0) • 63.4 (15.0)	• ^a^44 (35–55) • ^a^43 (34–53)	Meropenem	• CI: 3 g QD • BI: 1 g Q8H	34.14^d^	RCT, double blind	• Respiratory: 33/33 • Abdominal: 9.6/8.1 • Bloodstream: 9.6/5.1 • Uroinfection: 5.5/4.1 • Other: 11/12	• Gram-positive: 116/103 • Gram-negative: 246/222 • *Klebsiella* spp: 72/59 • *Pseudomonas* spp: 48/44 • *Escherichia coli*: 44/44 • *Acinetobacter* spp: 28/22 • *Enterobacter* spp: 13/15 • Other: 41/38	MIC determination	Not happened in both groups

ICU, intensive care unit; CI, continuous infusion; BI, intermittent bolus; LD, loading dose; MD, maintaining dose; RCT, randomized controlled trial; CNS, Central nervous system; SAE, serious adverse effect; MIC, minimal inhibition concentration; APCHE II, Acute Physiology and Chronic Health Evaluation II score; PTZ, piperacillin-tazobactam.

^a^Simplified acute physiology score II, calculated based on the patient characteristics, reason for intensive care admission, and physiological abnormalities. The core range is from 0 to 163; a higher score indicates a higher severity of disease and a higher risk of death.

^b^The median 24-h dose was 3 g.

^c^Definite as pathogen MIC to meropenem ≥ 1.5.

^d^Definite as pathogen susceptibility test showed resistance to carbapenem.

**Table 2:** Detailed quality assessment of included studies using the Cochrane risk-of-bias 2 tool.

**First Author**	**Year**	**Randomization process**	**Intervention adherence**	**Missing outcome data**	**Outcome measurement**	**Selective reporting**	**Overall RoB**
Chytra	2012	S^a^	L	L	S^b^	L	S
Dulhunty	2013	S^a^	L	L	L	L	S
Dulhunty	2015	S^a^	L	L	L	L	S
Abdual	2016	L	L	L	S^b^	L	S
Zhao	2017	L	L	L	L	L	L
Giacomo	2023	L	L	L	L	L	L

^a^The studies did not provide allocation concealment details.

^b^The study was an open-label study.

### 3.2 Primary outcome: continuous infusion does not decrease the mortality rate

**Figure 3:**
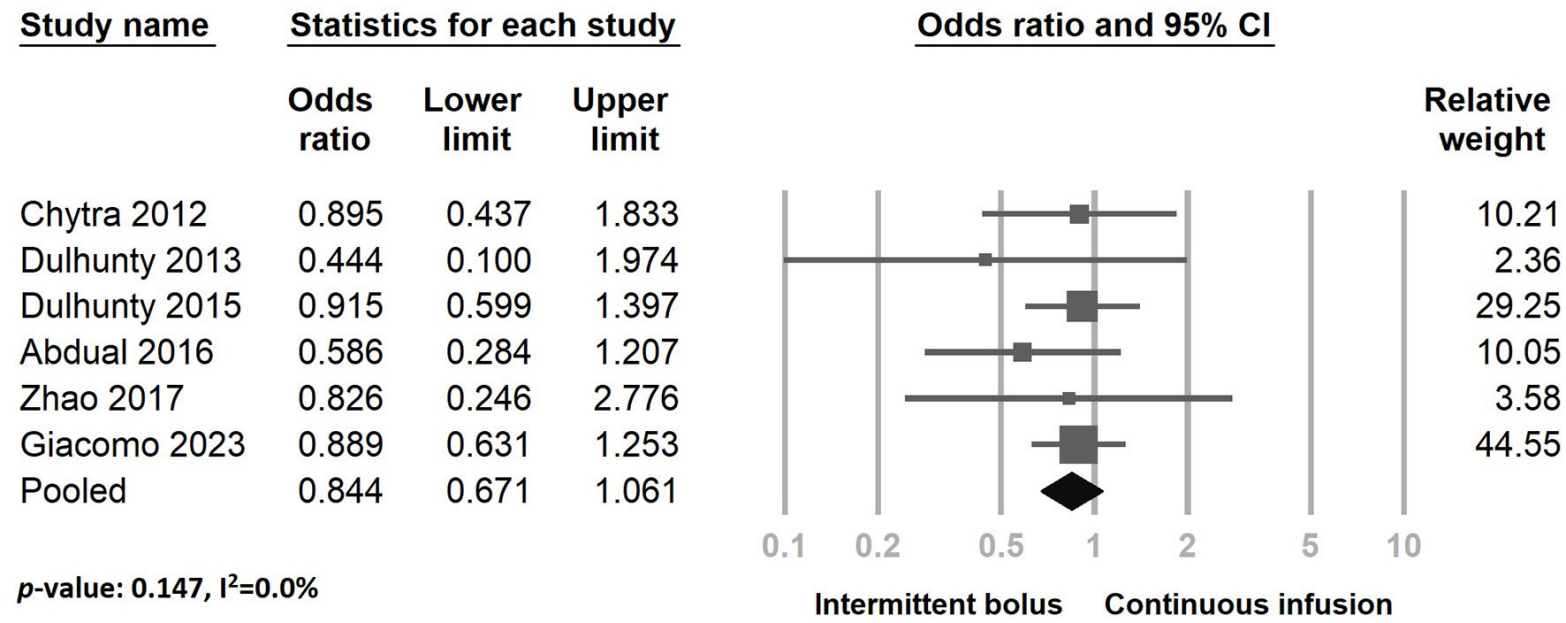
The forest plot of continuous infusion and intermittent bolus with mortality.

**Figure 4:**
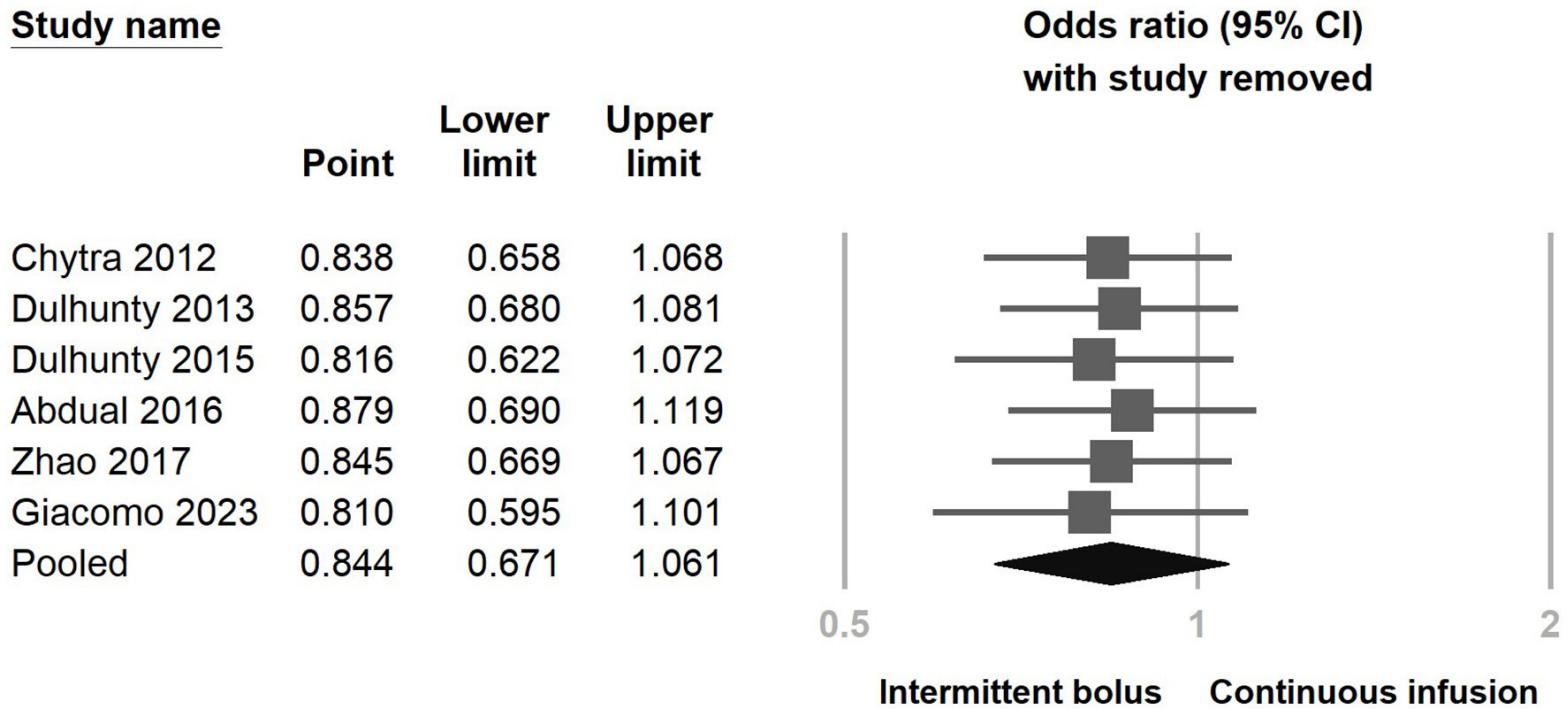
Sensitivity analysis of continuous infusion and intermittent bolus with mortality utilizing the one-study removal method.

**Figure 5:**
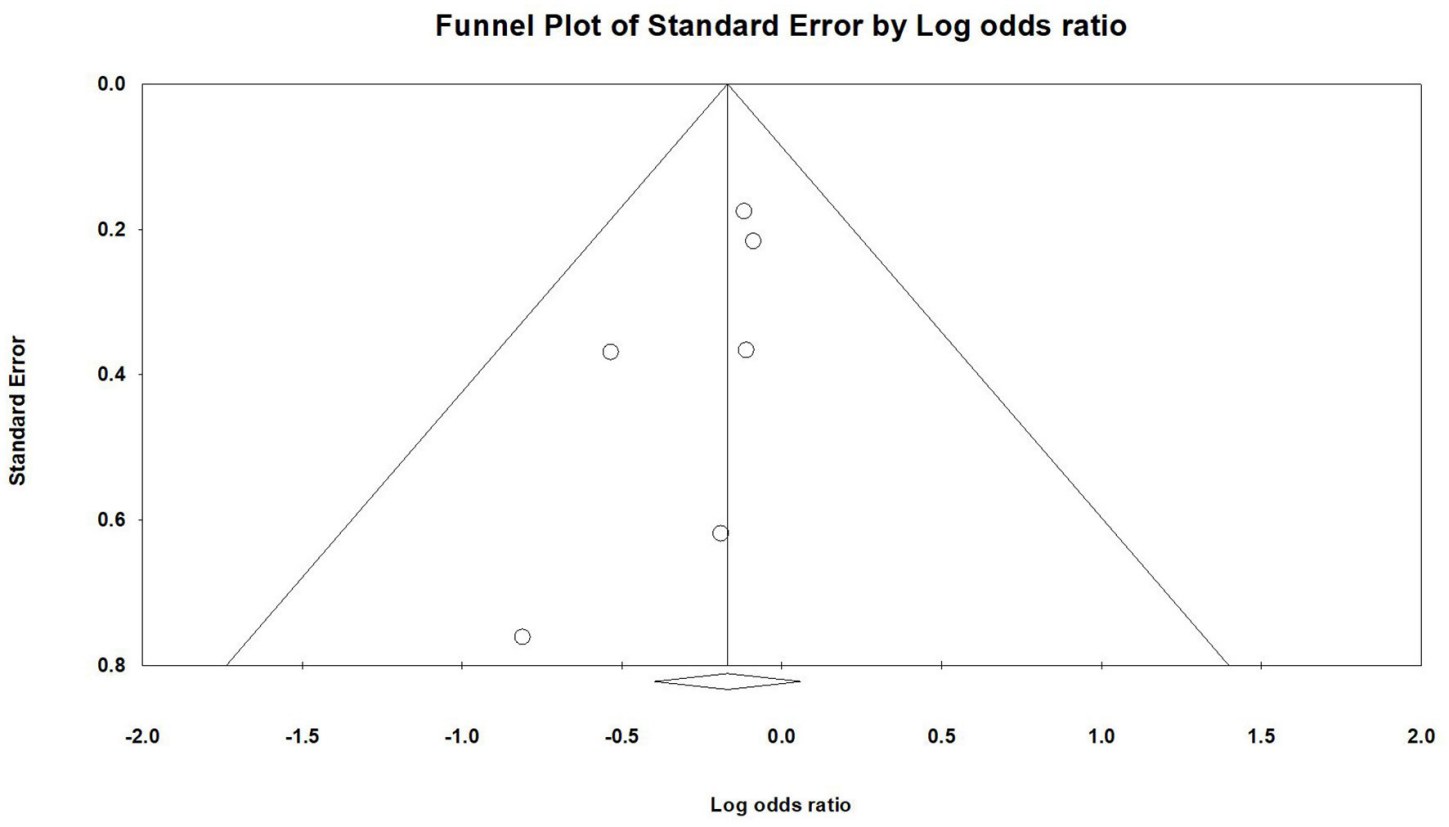
The funnel plot of continuous infusion and intermittent bolus with mortality.

Figure 3 illustrates the inclusion of six RCT studies, demonstrating that there was no significant difference in mortality rates between the continuous infusion and intermittent bolus groups (odds ratio: 0.844, 95% CI: 0.671–1.061, *P* = 0.147, *I*^2^ = 0.0%). To ensure the robustness of our findings, we performed a sensitivity analysis by removing one study, and the results remained unchanged (Figure 4). The presence of publication bias was examined using a funnel plot, as depicted in Figure 5 (Egger's *g* = 0.14014).

**Figure 6:**
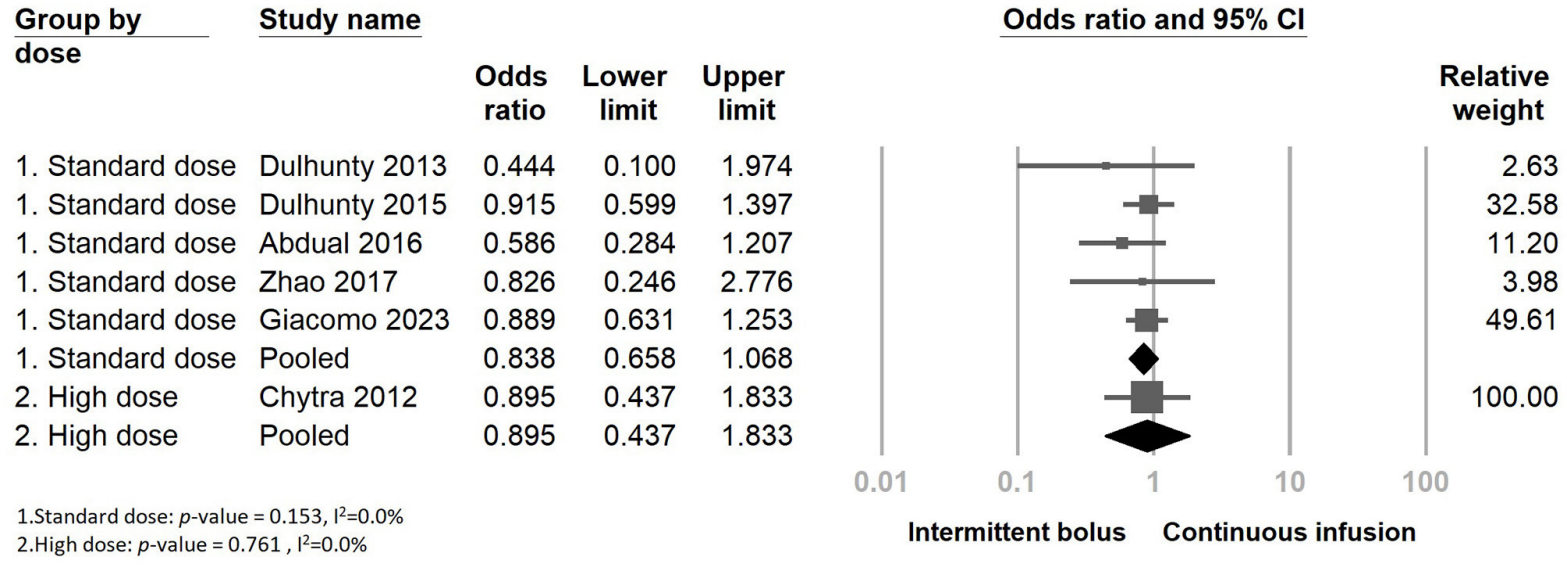
The forest plot of continuous infusion and intermittent bolus with mortality in the subgroup.

**Figure 7:**
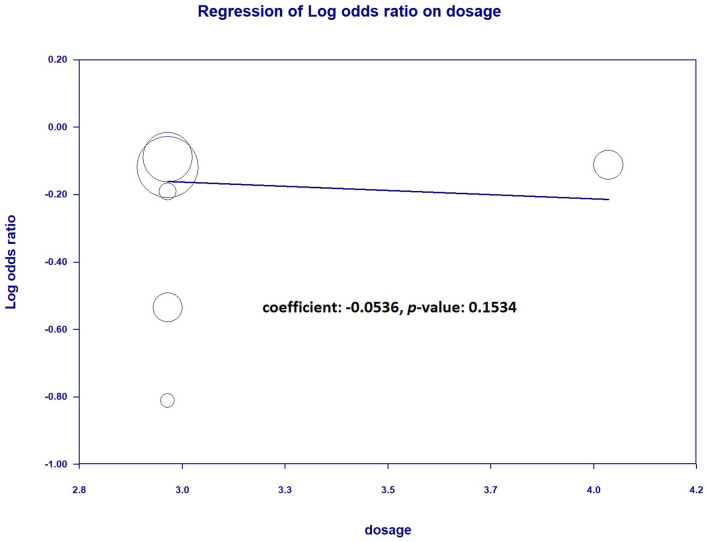
Regression between continuous infusion dosage and mortality.

Furthermore, we performed subgroup analyses depending on whether continuous infusion of other beta-lactam agents was included or not (Supplementary Figure S1). There was no statistically significant impact observed among the two subgroups regarding the continuous infusion of different antimicrobial agents. Additionally, we examined mortality rates within subgroups categorized by the meropenem dose (standard dose: 3 g/day, high dose: 4 g/day or more, adjusted according to eGFR). The results also indicated no decreasing mortality rates when continuous infusion was performed in the two subgroups (Figure 6). We further analyzed the dosage-dependent linear relationship between dosage and mortality rates, and the results showed no association (coefficient: −0.0536, *P* = 0.1534) (Figure 7).

### 3.3 Secondary outcomes: clinical success/improvement, bacteria eradication, length of stay in ICU (ICU LOS), ICU mortality, and adverse effects

**Figure 8:**
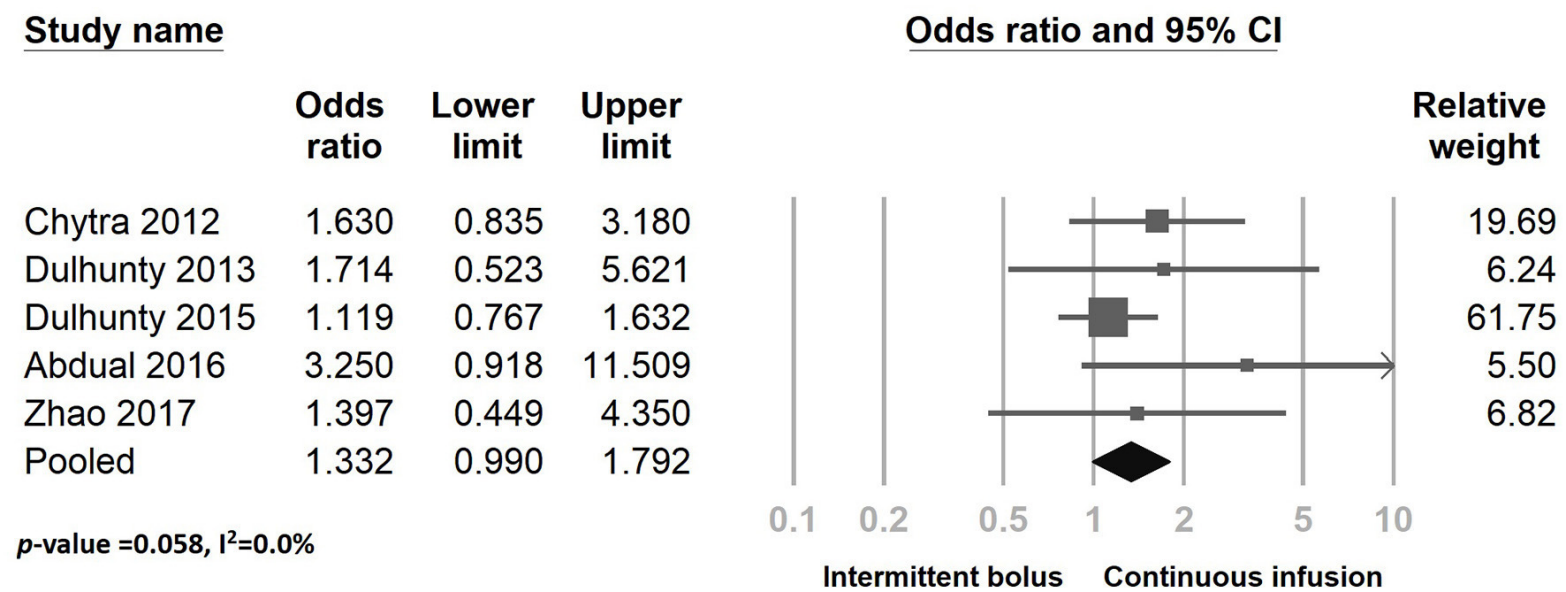
The forest plot of continuous infusion and intermittent bolus with clinical success.

**Figure 9:**
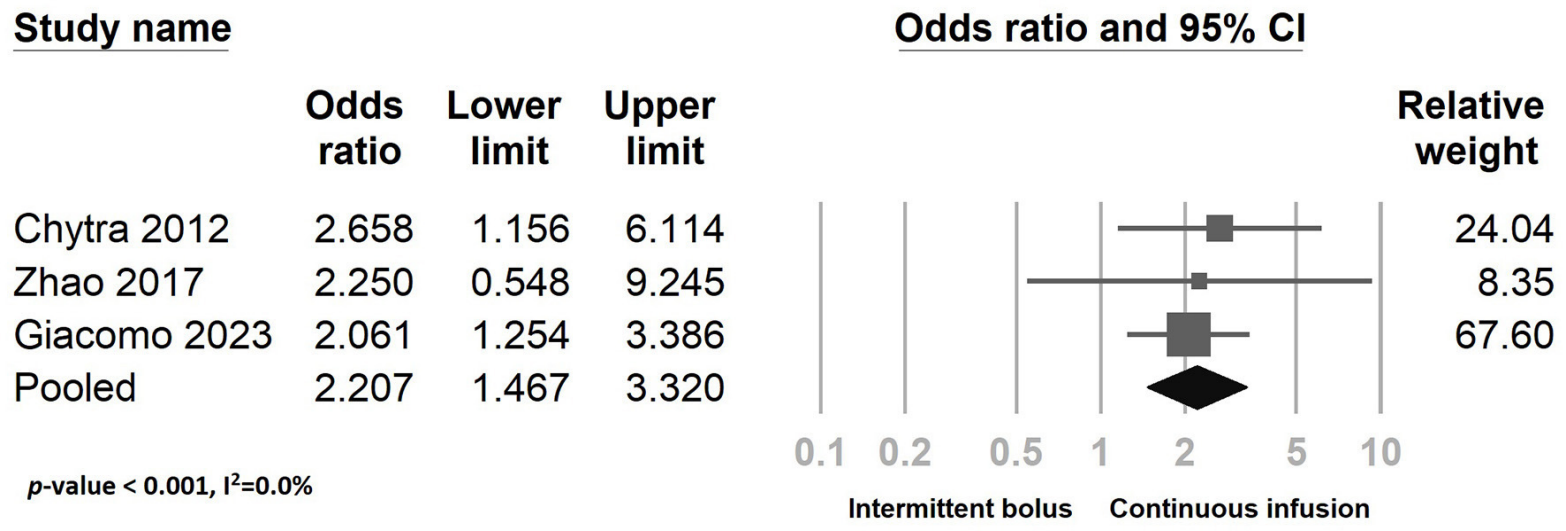
The forest plot of continuous infusion and intermittent bolus with bacteria eradication.

Figure 8 presents the clinical success outcome when comparing continuous infusion and intermittent bolus strategies. The results suggest that continuous infusion of meropenem significantly improves bacterial eradication rates (Figure 9) (odds ratio: 2.207, 95% CI: 1.467–3.320, *P* < 0.001, *I*^2^ = 0.0%). However, it does not reduce ICU LOS (Supplementary Figure S5) (odds ratio: 0.978, 95% CI: 0.647–1.478, *P* = 0.916, *I*^2^ = 0.0%) and ICU mortality (Supplementary Figure S7) (odds ratio: 0.825, 95% CI: 0.588–1.157, *P* = 0.265, *I*^2^ = 0.0%). We conducted a sensitivity test (Supplementary Figures S3, S4; Figures 7, 9) and examined the publication bias using a funnel plot (Supplementary Figures S10–S13). The sensitivity test yielded consistent results, and no significant publication bias was detected.

However, when analyzing the subgroup of meropenem alone and meropenem with other beta-lactam agents, the subgroup analysis of meropenem alone demonstrated a significant improvement in clinical success when continuous infusion was performed (Supplementary Figure S2) (odds ratio: 1.776, 95% CI: 1.051–2.999, *p*-value: 0.032).

**Figure 10:**
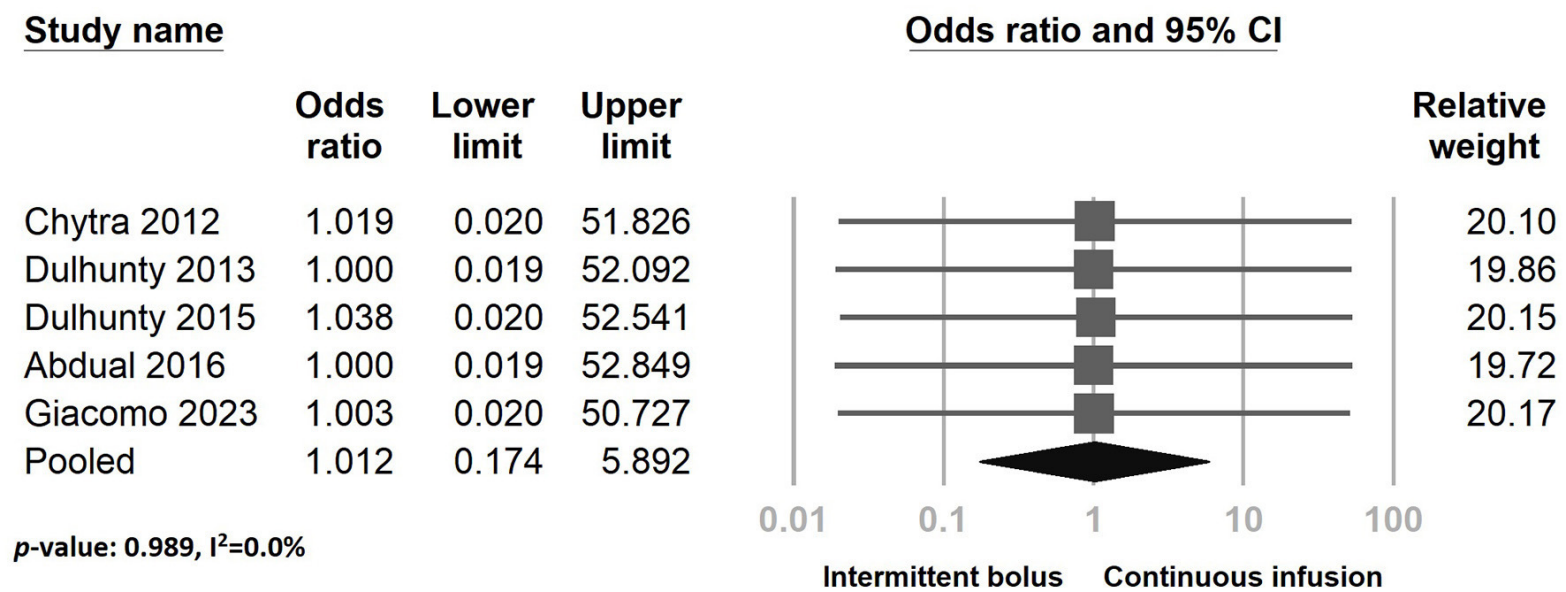
The forest plot of continuous infusion and intermittent bolus with serious adverse effects.

Regarding adverse effects, five studies reported no treatment-associated adverse events during the study period in both the continuous and intermittent bolus groups. However, in the Dulhunty 2015 study, four adverse events were reported during the study period. Our analysis indicated no significant difference between continuous infusion and intermittent bolus administration in adverse events (Figure 10) (odds ratio: 1.012, 95% CI: 0.174–5.892, *p*-value: 0.989, *I*^2^ = 0.0%). The sensitivity test and funnel plot are shown in Supplementary Figure S9 (Egger's *g* = 0.25718). The examination showed no significant publication bias (Supplementary Figure S14).

### 3.4 Secondary outcomes: the relationship between mortality/bacteria eradication and resistant pathogens when continuous infusion was performed

**Figure 11:**
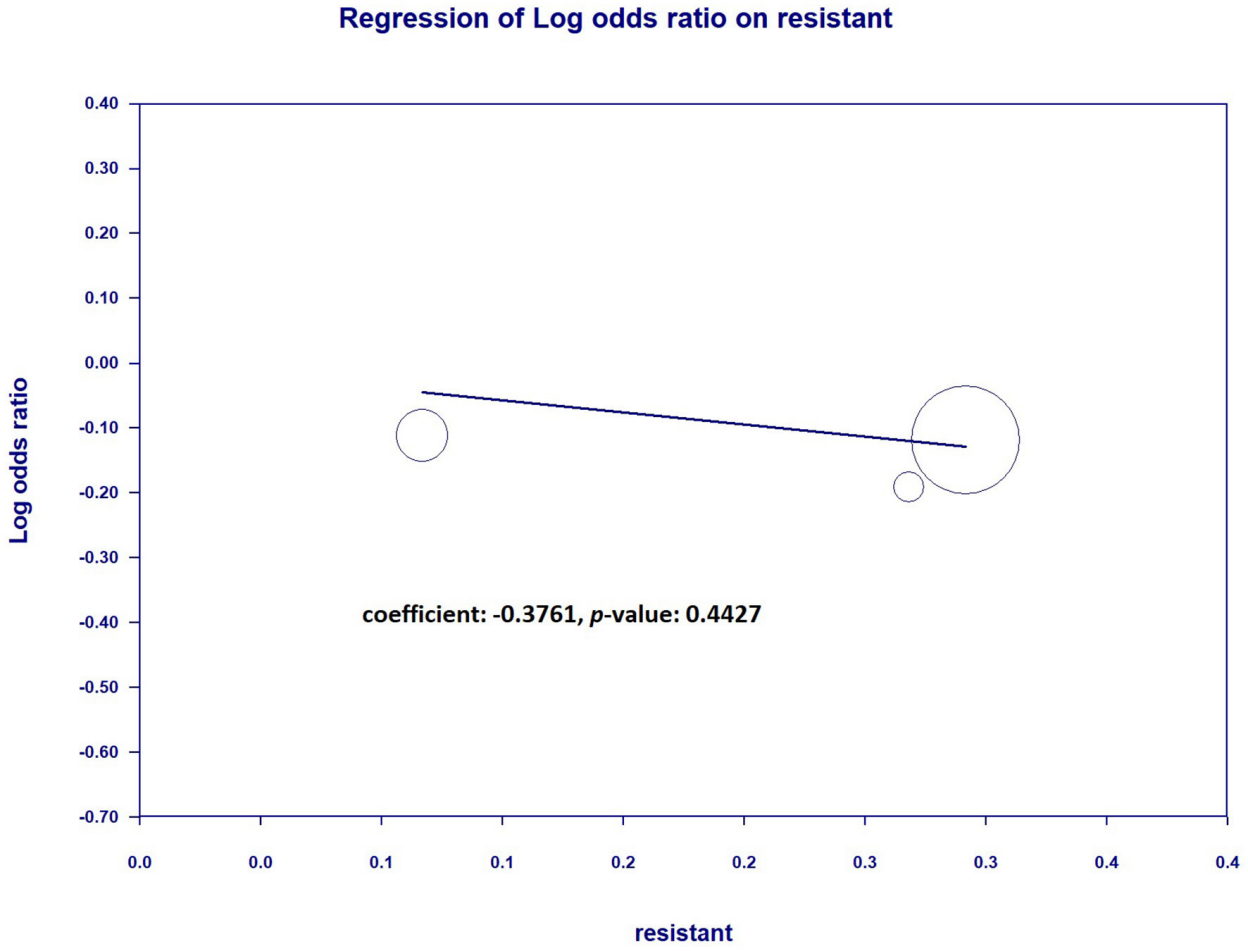
Regression between resistant pathogen (%) and mortality.

**Figure 12:**
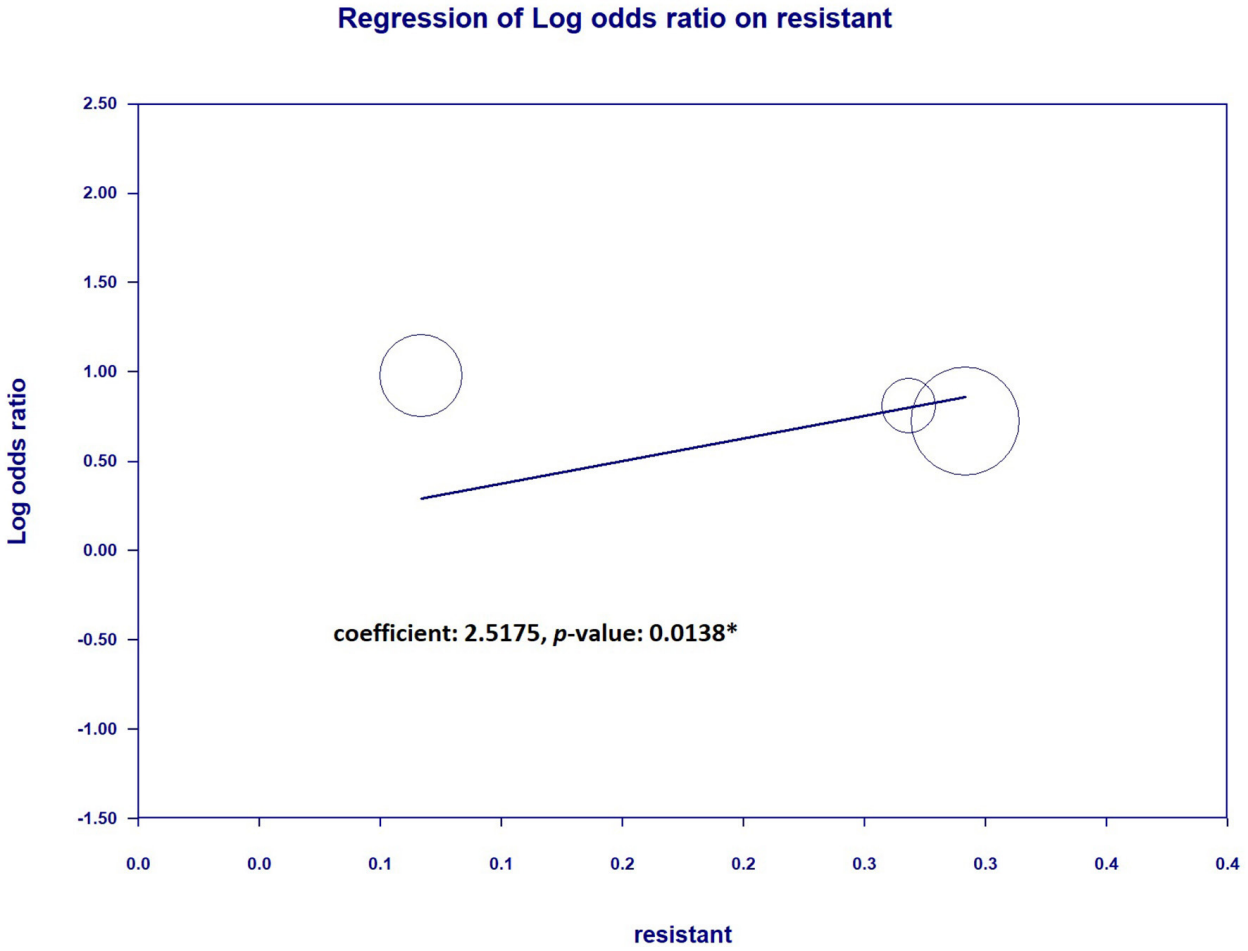
Regression between resistant pathogen (%) and bacterial eradication.

To further understand the impact on mortality and bacteria eradicated when continuous infusion of resistant pathogens was performed, we tested the meta-regression between the percentage of resistant pathogens and mortality or bacteria eradicated. The results showed that there was no significantly decreasing mortality when the cultures contained more resistant pathogens (coefficient: −0.3761, *P* = 0.4427) (Figure 11). However, the bacteria eradicated were significantly more resistant to pathogens when continuous infusion was performed (coefficient: 2.5175, *P* = 0.0138^*^) (Figure 12).

## 4 Discussion

In our meta-analysis study, there was no significant difference in the mortality rate, ICU LOS, ICU mortality, or adverse effects observed between continuous infusion and intermittent bolus administration of meropenem. However, the adoption of continuous infusion of meropenem resulted in a significant increase in both clinical success/improvement and bacterial eradication rates. The most interesting finding, meta-regression, showed that with more resistant pathogens, the continuous infusion could perform better bacterial eradicated effects.

Extended and continuous infusion strategies have gained wide acceptance in the administration of beta-lactam antibiotics due to their potent stable bactericidal effects (Yang et al., 2015). The effectiveness of bacterial eradication in beta-lactams is positively correlated with the duration of time that the drug concentration remains above the MIC. This is commonly measured as the percentage of the dosing interval during which the concentration of free drug exceeds the MIC, known as %fT > MIC (Berry and Kuti, 2022). Theoretically, the maximum bactericidal effect is achieved when the free drug concentration exceeds the pathogen's MIC. Carbapenem, being a broad-spectrum beta-lactam antibiotic used for treating multidrug-resistant pathogens, especially meropenem, has been widely considered for continuous infusion (Benavent et al., 2023). To optimize the antibacterial effect, the percentage of T > MIC should be higher than 30–50% between dosing intervals, depending on the bacterial species. Escalating the dose of meropenem or continuous infusion has been adopted to improve the %fT > MIC. However, despite the potential pharmacokinetics (PK) and pharmacodynamics (PD) advantages, it is unclear if improving %fT > MIC is associated with better clinical outcomes.

It was interesting to discover that, in contrast to previous studies, continuous infusion of beta-lactam antibiotics, particularly piperacillin-tazobactam, exhibited significantly superior efficacy (Hyun et al., 2022). When we conducted an independent analysis of continuous infusion of meropenem in a randomized control study, there was no significant difference observed in the mortality rates. To further investigate whether these results were influenced by varying daily dosages, we conducted subgroup analyses. Nonetheless, the mortality rate showed no significant difference between continuous infusion and intermittent bolus administration, both in the standard and high-dose groups. The mortality rate was not correlated with the dosage of meropenem.

Reflecting on previous studies regarding continuous infusion of meropenem, it can be observed that, while studies have shown clinical success or improvement with continuous infusion, they did not demonstrate reduction in mortality rates (Helmy et al., 2015). In a prospective randomized pilot study by Zhao in 2017, the author also examined PK data comparing continuous infusion and intermittent bolus administration. The results indicated that the trough concentration of meropenem in the continuous infusion group was 10-fold higher than that in the intermittent group for the first and third doses. Additionally, the concentration during ~40% of the dosing interval was twice as high in the continuous infusion group. These findings suggested that continuous infusion maintained a more stable therapeutic level during meropenem treatment. However, these effects did not correlate positively with the mortality rate of patients. Zhao's study demonstrated no significant difference in 28-day mortality rates between the two groups, as did our meta-analysis.

Previous studies have hypothesized that continuous infusion may offer benefits and increased potency against resistant pathogens. One of the studies described the rationale, principles, and dosage calculations for continuous infusion beta-lactam antibiotics, particularly focusing on their role in treating multidrug-resistant bacterial infections in patients undergoing continuous veno-venous hemofiltration (CVVH). The authors concluded that continuous infusion of beta-lactam antibiotics could be an effective treatment strategy for multidrug-resistant gram-negative bacteria infections in intensive care settings (Moriyama et al., 2009). Truong's 2022 study also investigated the impact of continuous meropenem infusion on resistant *Klebsiella pneumoniae* strains. This study utilizes PK data from ICU patients and collected *Klebsiella pneumoniae* isolates to develop feasible meropenem dosing regimens for treating infections caused by resistant *Klebsiella pneumoniae* strains (Truong et al., 2022). In a multicenter randomized controlled study by Dulhunty in 2015, similar results to Zhao's study were obtained, with no significant difference in the long-term 90-day mortality rate between the continuous infusion and intermittent bolus groups. However, upon closer observation of this study, it was found that only 2.5 vs. 14.0% of the pathogens in both the intervention and control groups were resistant pathogens. In another randomized open-label controlled trial by Chytra in 2012, which included a population with only 14.6% cultured-resistant pathogens, no significant difference in the mortality rate was also observed. Both RCTs indicate that continuous infusion may not be necessary for pathogens susceptible to the antibiotic, even in cases of severe infection with high APACHE II scores and ICU stays. To figure out the benefit subgroup of continuous infusion of meropenem, we performed meta-regression to find out the relationship between resistant pathogens and mortality or the bacterial eradication effect. The results showed that, when more resistant pathogens were included in the study, better bacterial eradication was found. It suggests that continuous infusion of meropenem may be beneficial in aiding the eradication of resistant pathogens. We postulated that, when treating wild-type pathogens, the MIC of meropenem is relatively low as compared to the drug-resistant isolates. Therefore, both the intermittent bolus and continuous infusion groups can reach the PK/PD target of %fT > MIC larger than 40%, which leads to no obvious clinical benefits in the continuous infusion group. However, for more drug-tolerant or drug-resistant strains, the traditional intermittent bolus group may not reach the PK target in some patients, while the continuous infusion group can maintain a more stable drug level above the MIC (Truong et al., 2022). Therefore, a trend toward improved clinical success rates and statistically improved microbial eradication rates could be observed in the drug-resistant pathogen subgroup analysis. In our future study, we plan to gather clinical data from patients who have been infected with resistant bacteria and treated with continuous infusion of meropenem. We will then use these data to analyze and develop a PK and PD model, aiming to understand the relationship between %fT > MIC and the outcomes of the treatment.

Another hypothesis suggesting an advantage of continuous infusion for beta-lactam antibiotics is the provision of a stable therapeutic level, which may help prevent the emergence of resistant pathogens (Buck et al., 2005). Extended and continuous infusion methods offer less time below the MIC, which is believed to be a period during which bacteria can re-grow. Continuous infusion ensures that the serum drug concentration remains consistently above the MIC (Craig, 2003). Rapid bacterial killing can also reduce the chances of pathogens acquiring new genetic elements from one another. In previous *in vitro* studies, maintaining a stable antibiotic level has been shown to prevent bacterial growth and resistance (Li et al., 2014). However, further studies are needed to provide more evidence for the benefits of this theory. In the Giacomo 2023 study, the primary outcome aimed to examine the emergence of resistant pathogens (defined as pan-drug resistant or resistant to all but one or two drug classes) between continuous infusion and intermittent bolus infusion groups. The results revealed no significant difference between the two groups. Although *in vitro* studies hypothesize that a stable drug concentration might reduce the likelihood of pathogens developing resistance, the same result was not confirmed in the *in vivo* study.

Safety concerns associated with continuous infusion method have always been a prominent issue. Extended and continuous infusion strategy have the potential to maintain stable drug levels in the bloodstream and tissues, which could alleviate concerns regarding drug toxicity compared to intermittent dosing (Manning et al., 2014). In our meta-analysis study, which comprised five studies, none of the studies reported serious adverse events occurring in either the continuous infusion or intermittent bolus groups. In general, beta-lactam antibiotics are well-tolerated compared to other classes of antibiotics, and it appears that continuous infusion strategies demonstrate a similar level of safety as traditional intermittent bolus strategies (Chiriac et al., 2017). In our meta-analysis study, no significant difference was observed in the occurrence of adverse events between continuous infusion and intermittent bolus administration of meropenem.

Our study has several limitations. First, all the included studies allowed for the use of additional therapeutic antibiotics based on the patients' condition, which introduces bias to the assessment of patient mortality. Second, the small sample sizes and variations in the loading dose of meropenem among different RCTs could also impact the mortality rates observed. Third, the definitions and criteria for clinical success and improvement varied across the studies, which could contribute to differences in the study outcomes. Finally, most of the mortality rates were analyzed at 28 days, but two studies measured the mortality rates at 90 days, which could also affect the overall mortality rate results.

## 5 Conclusion

In our meta-analysis study, we found no significant difference in the mortality rate, ICU LOS, ICU mortality, or adverse events between continuous infusion and traditional intermittent bolus strategies of meropenem. Despite using a high dose (4 g) of meropenem through continuous infusion, there was no decrease in mortality rates compared to intermittent bolus administration. However, our study found that continuous infusion of meropenem may lead to better bacterial eradication effects, especially in resistant pathogens. We observed enhanced microbiological eradication rates when utilizing continuous infusions of meropenem for managing infections, particularly those caused by drug-resistant pathogens. There was also a trend indicating improved clinical success rates with this approach. Thus, continuous infusion of meropenem may offer a safe and a potentially effective treatment strategy for patients with drug-resistant infections.

## Data availability statement

The raw data supporting the conclusions of this article will be made available by the authors, without undue reservation.

## Author contributions

M-YA: Conceptualization, Data curation, Formal analysis, Methodology, Project administration, Writing – original draft. W-LC: Investigation, Methodology, Writing – original draft. C-YL: Conceptualization, Formal analysis, Project administration, Supervision, Writing – original draft, Writing – review & editing.
